# Zonula occludens‐1 distribution and barrier functions are affected by epithelial proliferation and turnover rates

**DOI:** 10.1111/cpr.13441

**Published:** 2023-03-14

**Authors:** Keisuke Imafuku, Hiroaki Iwata, Ken Natsuga, Makoto Okumura, Yasuaki Kobayashi, Hiroyuki Kitahata, Akiharu Kubo, Masaharu Nagayama, Hideyuki Ujiie

**Affiliations:** ^1^ Department of Dermatology, Faculty of Medicine and Graduate School of Medicine Hokkaido University Sapporo Japan; ^2^ Department of Dermatology Gifu University Graduate School of Medicine Gifu Japan; ^3^ Research Institute for Electronic Science Hokkaido University Sapporo Japan; ^4^ Department of Physics, Graduate School of Science Chiba University Chiba Japan; ^5^ Division of Dermatology, Department of Internal Related Kobe University Graduate School of Medicine Kobe Japan; ^6^ Department of Dermatology Keio University School of Medicine Tokyo Japan

## Abstract

Zonula occludens‐1 (ZO‐1) is a scaffolding protein of tight junctions, which seal adjacent epithelial cells, that is also expressed in adherens junctions. The distribution pattern of ZO‐1 differs among stratified squamous epithelia, including that between skin and oral buccal mucosa. However, the causes for this difference, and the mechanisms underlying ZO‐1 spatial regulation, have yet to be elucidated. In this study, we showed that epithelial turnover and proliferation are associated with ZO‐1 distribution in squamous epithelia. We tried to verify the regulation of ZO‐1 by comparing normal skin and psoriasis, known as inflammatory skin disease with rapid turnover. We as well compared buccal mucosa and oral lichen planus, known as an inflammatory oral disease with a longer turnover interval. The imiquimod (IMQ) mouse model, often used as a psoriasis model, can promote cell proliferation. On the contrary, we peritoneally injected mice mitomycin C, which reduces cell proliferation. We examined whether IMQ and mitomycin C cause changes in the distribution and appearance of ZO‐1. Human samples and mouse pharmacological models revealed that slower epithelial turnover/proliferation led to the confinement of ZO‐1 to the uppermost part of squamous epithelia. In contrast, ZO‐1 was widely distributed under conditions of faster cell turnover/proliferation. Cell culture experiments and mathematical modelling corroborated these ZO‐1 distribution patterns. These findings demonstrate that ZO‐1 distribution is affected by epithelial cell dynamics.

## Graphical abstract

Left: schematic diagram of tight junction (TJ) distribution in the slow turnover/proliferation tissues, including normal skin, oral lichen planus (OLP) and mitomycin C (MMC)‐treated mice. Right: schematic diagram of TJ distribution in the rapid turnover/proliferation tissues, including normal buccal mucosa, psoriasis and imiquimod‐treated mice.

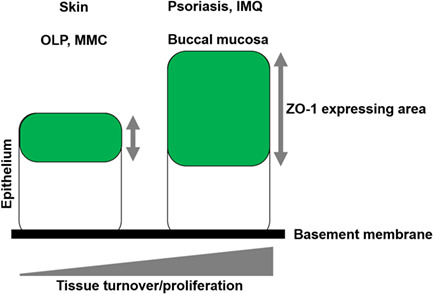

## 1 INTRODUCTION

Cellular adhesion determines cell polarity and is supported by the dynamics of membrane proteins, cytoskeletons and signal transduction pathways. Multicellular organisms rely on cellular adhesion (e.g., the lining of epithelial cells) to differentiate themselves from the external environment. In vertebrates, the four intercellular junctional complexes are the following: adherens junctions (AJs), gap junctions, desmosomes and tight junctions (TJs).

The skin functions as a physical barrier to the outside world.^1^ TJs are responsible for key barrier functions, such as regulating the passage of substances through paracellular spaces and controlling the diffusion of water and ions between the cells and the external environment.^2^, ^3^, ^4^

A recent study demonstrated that TJs are found in the second layer of the stratum granulosum (SG2) in mouse ear skin^5^ and human skin.^6^ They are complex structures that are composed of more than 40 proteins,^7^ including transmembrane proteins (i.e., occludin,^8^ tricerullin,^9^ angulin,^10^ JAM family^11^ and claudin family^12^, ^13^, ^14^) and intracellular scaffold proteins, such as the zonula occludens (ZO) family.^15^, ^16^ The claudin family proteins bond tightly to each other and organize TJ strands.^14^

During early differentiation, the TJ scaffold protein ZO‐1 is also expressed in AJs.^17^ We have recently shown that the distribution pattern of ZO‐1 differs between the buccal mucosa and skin.^18^ However, the underlying mechanism of these differences in the distribution pattern is unclear. In this study, we explored the role of epithelial cell dynamics in the regulation of ZO‐1 expression. We employed human tissue samples, pharmacological treatments of murine skin and mathematical modelling to show that ZO‐1 distribution is affected by the rate of epithelial proliferation and turnover.

## 2 MATERIALS AND METHODS

### 2.1 Human tissue samples

All human studies were approved by the Institutional Review Board of the Graduate School of Medicine, Hokkaido University (ID: # 14‐063, # 15‐025). This study was carried out in accordance with the Declaration of Helsinki. Participants or their legal guardians provided written informed consent. Samples of normal skin (*N* = 3), psoriatic skin (*N* = 3), normal buccal mucosa (*N* = 3) and oral lichen planus (OLP) tissue (*N* = 3) were collected and used in the study.

### 2.2 Animals

C57BL/6 mice were purchased from CLEA, Japan, Inc. All animal protocols were approved by the Animal Ethics Review Board of Hokkaido University (#15‐0166 and #19‐0164) and conform to the National Institutes of Health guidelines.

### 2.3 Mitomycin C treatment

Mitomycin C treatment in mice^19^ was performed with some modifications. Mitomycin C solution (Fuji Film Co., Ltd., Japan) was adjusted to a concentration of 2 mg/mL. C57BL/6J mice were intraperitoneally injected with a 5–10 mg/kg mitomycin C solution, euthanized after 10 days and the oral mucosal tissue was collected. Body weights were measured before each procedure.

### 2.4 Mouse model of psoriasis

Psoriasis‐like skin in mice was induced using a modified protocol described in a previous report.^20^ Briefly, 5% imiquimod (IMQ) cream (Maruho Co., Ltd., Japan) was applied daily to the dorsal surface of the ears of 8‐ to 12‐week‐old male C57BL/6J mice for 4 days, after which the skin samples were collected. Ear thickness was measured daily using a pocket thickness gauge (Mitutoyo Europe GmbH, Germany), and body weight was recorded before the animals were sacrificed.

### 2.5 Cell culture

Immortalized human keratinocytes derived from infant foreskin (KerCT®) were obtained from ATCC (VA). KerCT cells were cultured in the Keratinocyte Growth Medium (KGM, Lonza Bioscience, Switzerland) at 37°C, 5% CO_2_. EGF (epidermal growth factor, AF‐100‐15, PeproTech, NJ) was used as a reagent during the incubation period (at a final concentration of 10 ng/mL).

### 2.6 Histology

Human and mouse tissue samples were fixed with formalin and embedded in paraffin following dehydration. For morphological analysis, deparaffinized sections were stained with haematoxylin and eosin (H&E) by conventional methods.^21^ Images of H&E‐stained sections were captured with a BZ‐9000 microscope (Keyence, Tokyo, Japan).

### 2.7 Immunofluorescence

Skin and buccal mucosa specimens were frozen in an optimal cutting temperature (OCT) compound (Cryomount I, 33351; Muto Pure Chemicals, Japan) and sectioned with a cryostat (Leica CM1950, Leica Biosystems, Germany). To prepare horizontal‐sliced sections, specimens were embedded horizontally in the OCT compound, and 5 μm‐thick sections were prepared. Frozen sections were incubated with primary antibodies: rabbit anti‐ZO‐1 (dilution 1:200 in PBS, ab2168; Abcam, UK), rabbit anti‐claudin‐1 (dilution 1:200 in PBS, ab15098; Abcam, UK), rabbit anti‐occludin (dilution 1:100 in PBS, 71‐1500; Invitrogen, CA), mouse anti‐keratin14 (dilution 1:200 in PBS, ab7800; Abcam, UK), rabbit anti‐keratin10 (dilution 1:500 in PBS, 905404; BioLegend, CA), rabbit anti‐cleaved caspase‐3 (dilution 1:100 in PBS, #9661 S, Cell Signalling Technology, MA) or anti‐Ki‐67 (dilution 1: 200, NB600‐152, Novus Biologicals) at 37°C for 60 min. The sections were subsequently incubated with Fluorescein isothiocyanate (FITC)‐conjugated goat anti‐rabbit IgG H + L (dilution 1:200 in PBS, 234; MBL), alexa 488‐conjugated anti‐mouse IgG3 (dilution 1:1000 in PBS) or alexa 568‐conjugated anti‐rabbit IgG (dillution 1:1000 in PBS) at 37°C for 60 min and mounted in a Pharma Fluor aqueous mounting medium (TA‐006‐FM; Thermo Fisher Scientific, MA). The stained immunofluorescent samples were observed with a BZ9000 microscope.

### 2.8 Whole‐mount staining

Whole‐mount staining was carried out according to procedures described in the literature, with modifications.^18^, ^22^ Normal mouse buccal mucosa was fixed in 95% ethanol at room temperature for 60 min. Before peeling the epithelial sheets from the lamina propria, the tissue was soaked in a solution of Dispase I (386‐02271; Fujifilm, Tokyo, Japan) at a concentration of 1000 PU/mL in PBS with 1.8 mM calcium (14040‐133; Thermo Fisher Scientific) at 4°C overnight. After incubation, the epithelial sheets were mechanically peeled and floated in a solution of 5% bovine serum albumin (final concentration, 5%) and 0.25% triton X‐100 (final concentration, 0.125%) at 4°C for 18 h. The samples were subsequently washed with PBS and incubated with goat anti‐human ZO‐1 Ab (dilution 1:200 in PBS, PA5‐19090; Thermo Fisher Scientific) in 5% BSA at 4°C overnight. In the following day, the samples were washed with PBS and incubated with Alexa Fluor 488‐conjugated donkey anti‐goat IgG H&L Ab (dilution 1:200 in PBS, A‐11055; Thermo Fisher Scientific) for 60 min at room temperature. After washing with PBS, the samples were mounted with a Mowiol solution (2.4 g Mowiol [81381‐250G; Sigma‐Aldrich, St. Louis, MO], 6.0 g glycerol, 1.6 mL 1.5 M tris‐HCL [pH 8.8] and 16.4 mL distilled water). Confocal microscopic images were taken with an FV1000 (Olympus, Japan).

### 2.9 Immunohistochemistry

Formalin‐fixed and paraffin‐embedded 3 μm‐thick sections were deparaffinized with xylene and serially diluted with ethanol. The sections were washed with Tris‐buffered saline (TBS) and subsequently treated with Dako EnVision FLEX Target Retrieval Solution (pH 9.0) (K8004, DAKO, Denmark) at 95°C for 20 min to retrieve the antigen. The sections were blocked with H_2_O_2_ and reacted with anti‐Ki‐67 antibody or anti‐loricrin antibody (dilution 1:2000, ab198994, Abcam) for 30–60 min, followed by incubation with horseradish peroxidase (HRP)‐labelled anti‐rabbit IgG polyclonal antibody (Histofine Simple Stain MAX‐PO (MULTI), 424151, Nichirei Bioscience, Japan) for 30 min. Next, the sections were reacted with DAB (3,3’‐diaminobenzidine, 425011, Nichirei Bioscience, Japan) for 5 min for the colour development. Haematoxylin staining was performed as a counterstain, and the reaction was carried out for 1.5 min.^23^

### 2.10 Immunoblotting

Cell lysates were prepared as in the previous literature.^24^ Anti‐ZO‐1 antibody (dilution 1: 5000, ab216880, Abcam) and HRP‐labelled anti‐Rabbit IgG (dilution 1: 5000, Invitrogen) were used.

### 2.11 Quantitative real‐time polymerase chain reaction (qPCR)

Total RNA extraction and cDNA generation were processed as in the previous report.^24^ All primers (Table S1) were obtained from Integrated DNA Technologies (IDT, Coralville, IA). Target gene expression levels were normalized to the expression level of the glyceraldehyde 3‐phosphate dehydrogenase (GAPDH) gene.

### 2.12 Skin equivalent model (SEM)

According to the manufacturer's protocol with minor modifications, Ker‐CTs were cultured on Nunc™ Polycarbonate Cell Culture Inserts (0.4‐μm pore size, 140668, Thermo Fisher Scientific) for 2–3 days with the CnT‐PR medium. After 48 h, the medium was changed from Cnt‐PR to CnT‐PR‐3D (#CnT‐ PR‐3D, Cellntec, Switzerland), and the cells were incubated for an additional 2 days. Next, the medium in the insert was discarded, and the cell sheets were cultured for 12 days, with the medium changed three times a week.^25^

### 2.13 Permeation assay

The TJ barrier function was assessed in vivo using a previously described biotinylation technique^26^, ^27^, ^28^ with some modifications. First, 50 μL of 10 mg/mL EZ‐Link Sulfo‐NHS‐LC‐Biotin (21355; Thermo Fisher Scientific) in PBS containing 1‐mM CaCl_2_ (C‐34006; PromoCell, Heidelberg, Germany) was injected by Myjector syringe (SS‐05M2913; Terumo Corporation, Tokyo, Japan) into the dorsal ear of the C57BL/6J mouse under anaesthesia. After 60 min, we sacrificed the mouse and collected ear samples. In the 3D model, the insert was turned upside down and the tracer solution was applied to the bottom side. Sixty‐minutes later, cell sheets were collected. We soaked in vitro and in vivo samples in a frozen section embedding agent (Cryomount I, 33351; Muto Pure Chemicals) and sectioned them with a cryostat (Leica CM1950, Leica Biosystems). The staining procedure was performed as described above, except that streptavidin Texas Red (dilution 1:200 in PBS 189738; Merck, Darmstadt, Germany) was additionally mixed into the solution of secondary antibodies. Fluorescence microscopic images were taken (BZ9000, BioRevo; Keyence).

### 2.14 Paracellular flux measurement

Ker‐CTs were cultured on Nunc™ Polycarbonate Cell Culture Inserts by the method described above. Fluorescent conjugated dextrans of various molecular weights was applied to the apical side of the insert, and the fluorescence intensity of the FITC‐dextran solution that passed through the cell sheet was measured with a protocol modified from previous reports.^29^

Two fluorescence‐conjugated tracers were applied to the apical side: 200 μM fluorescein (332.31 g/mol) (065‐00252, Fujifilm) and 4‐kDa FITC dextran (FD4, 60842‐46‐8; Sigma‐Aldrich). The volumes of the medium in the apical and basal chambers were 100 and 1000 μL, respectively. The chambers were incubated for 1 h. The tracer fluorescence intensities of the apical and bottom chambers were measured using the SpectraMax Paradigm Multi‐Mode Microplate Reader (Molecular Devices, San Jose, CA). The ratio of tracer fluorescence intensities to the fluorescence of the bottom side was calculated based on the apical side; ratio = Log_10_ (intensity [bottom]/intensity [apical]). The ratio shows a negative value, and the closer the value of the ratio is to 0, the more dye passes through the cell sheet.

### 2.15 Image analysis

Image analyses were performed with ImageJ NIH (Bethesda, MD; https://imagej.nih.gov/ij/) by preparing a plug‐in. In the plug‐in, an enhanced image was prepared by extracting the maximum value within a distance of 10 μm from each pixel. From the resulting enhanced image, the ZO‐1 intensity profile was obtained by averaging the brightness along a 40 μm line parallel to the horizontal aspects of the epidermis as a function of the depth. In the profile, the length of the region where the brightness was higher than 75% of the maximum brightness was set as ZO‐1, whereas the total length corresponding to the epithelial layers was set as *E*. The ZO‐1/E ratio was adopted to evaluate the expression of ZO‐1.

### 2.16 Mathematical modelling

We used a mathematical model for simulating 3D epidermal dynamics,^30^ which calculated proliferation, migration and differentiation. Proliferation was controlled by a cell‐division period T assigned to each cell in the stratum basale. Cell migration was computed by solving the equation of motion, where each cell was considered to be a spheroid. Differentiation into the stratum spinosum (SP), SG and SC was controlled by an inner variable S, which increased in time with the rate α. The SG cells were classified into SG1, SG2A, SG2B and SG3,^5^ and defined as follows: an SP cell became an SG3 when its value of S crossed the threshold S1*, and it was in contact with at least one SG2A cell. An SG3 cell became an SG2A when it was in contact with at least one SG2B cell. The subsequent process from SG2B to SG2A and from SG2A to SG1 was controlled by the threshold values of S2* and S3*, respectively, with S1* < S2* < S3*. We regarded SG2 cells (SG2A and SG2B) as expressing ZO‐1.

In the simulation used for this study, the basement membrane and the dermis were treated as a flat surface, whereas in the original model, they were deformable and undulating.^30^ All other conditions for the simulation were the same as that described in the reference.^30^ To investigate the effects of proliferation and turnover, we introduced an acceleration ratio *λ*, which changed the cell division period T to T/λ and the differentiation rate α to *λ*α, thereby accelerating both proliferation and turnover *λ* times as fast when *λ* > 1. The thickness of the ZO‐1 distribution was defined as follows: first, the horizontal region was divided into equally spaced 4 × 4 subregions, and the maximum vertical distance among cells was computed in each subregion. These distances were subsequently averaged across all subregions.

### 2.17 Statistical analysis

Statistical analysis was performed using Sigma plot® (ver14.5, HULINKS, Japan). *p*‐values were determined using the Student's *t*‐test if they were homoscedastic or Welch's *t*‐test if they were not homoscedastic. A *p*‐value <0.05 was considered statistically significant. The values are presented as the means ± standard deviations (SD).

## 3 RESULTS

### 3.1 Lower dispersal of ZO‐1 with slower proliferation and turnover in inflammatory oral mucosa

ZO‐1 is more dispersed in buccal mucosa than in skin^18^ (Figures S1 and S2). To further elucidate distinct ZO‐1 dispersal patterns, we focused on the cell dynamics in each type of epithelium. The turnover period in the buccal mucosa is typically 3.2–5.8 days,^31^ while that in the skin is 40–56 days.^32^ We hypothesized that this difference in the tissue turnover period could account for the distinct ZO‐1 distribution patterns observed in each epithelium type. To prove this hypothesis, we first investigated human buccal mucosa samples of OLP, in which the turnover period is pathologically prolonged due to the damage caused to basal keratinocytes by inflammation.^33^

**FIGURE 1:**
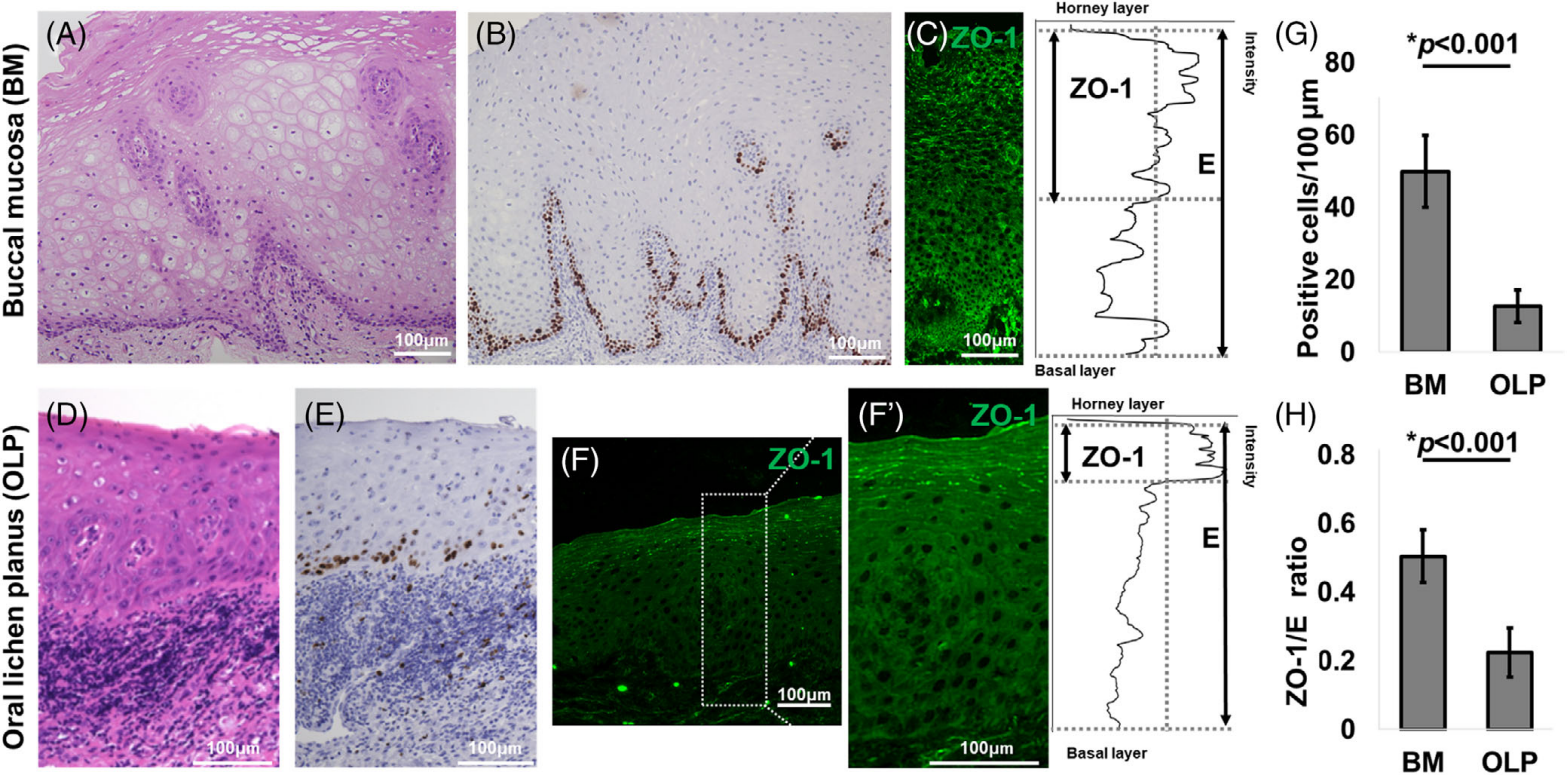
Zonula occludens‐1 (ZO‐1) distribution in oral lichen planus (OLP). (A) HE staining of normal human buccal mucosa (BM). Scale bar: 100 μm. (B) Ki‐67 staining of normal human BM. Scale bar: 100 μm. (C) Left: ZO‐1 immunofluorescent staining of normal human BM. Scale bar: 100 μm. Right: ZO‐1 intensity. E: Full epithelial layer. (D) HE staining of OLP. Scale bar: 100 μm. (E) Ki‐67 staining of OLP. Scale bar: 100 μm. (F) ZO‐1 immunofluorescent staining of OLP. Scale bar: 100 μm. (F’) Left: enlarged image of the white square of (F). Scale bar: 100 μm. Right: ZO‐1 intensity of the left panel. (G) The number of Ki‐67‐positive cells per 100 μm. (H) Relative ZO‐1 area to epithelial layers.

The thickness of the mucosal epithelium was reduced in OLP samples (Figure 1D) compared with that of normal buccal mucosa (Figure 1A) (664 vs. 195 μm on average). In accordance with the thinner epithelium, the number Ki‐67‐positive cells was reduced in OLP (Figure 1E,G) compared to that in normal buccal mucosa (Figure 1B). We then compared the ZO‐1 distribution pattern in OLP and normal buccal mucosa. ZO‐1 was dispersed in the upper half of the epithelium in normal human buccal mucosa (Figure 1C,H) and in normal mouse mucosa (Figures S1b and S2). In contrast, ZO‐1 was confined to the uppermost part of the epithelium in OLP (Figure 1F,F’ and 1H), which recapitulates skin ZO‐1 (Figure S1a).

### 3.2 Lower dispersal of ZO‐1 in mitomycin C‐treated oral mucosa

**FIGURE 2:**
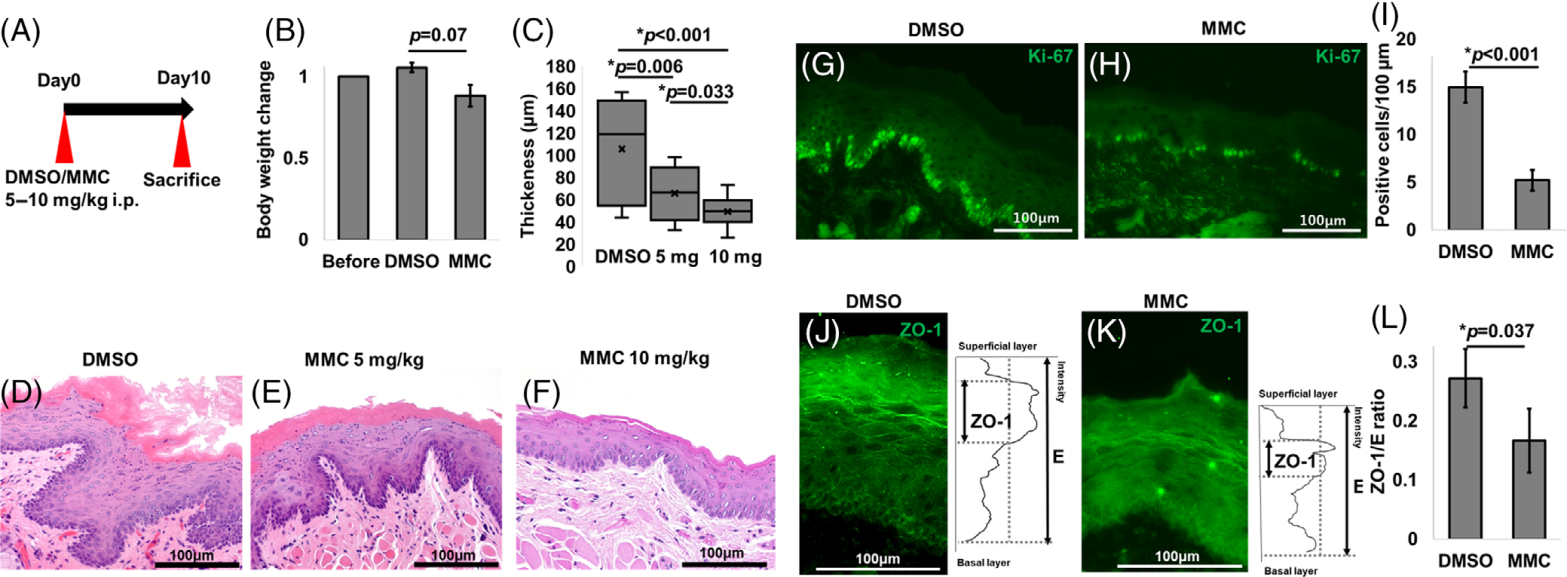
Zonula occludens‐1 (ZO‐1) distribution in mitomycin C (MMC)‐treated murine buccal mucosa (BM). (A) Schematic diagram of MMC treatment. (B) Body weight (BW) before and after MMC treatment. (C) Thickness of the BM after MMC treatment. (D) HE staining of the BM after Dimethyl sulfoxide (DMSO) treatment. Scale bar: 100 μm. (E) HE staining of the BM after MMC 5 mg/kg treatment. Scale bar: 100 μm. (F) HE staining of the BM after MMC 10 mg/kg treatment. Scale bar: 100 μm. (G) Ki‐67 immunofluorescent staining of the BM after DMSO treatment. Scale bar: 100 μm. (H) Ki‐67 immunofluorescent staining of the BM after MMC 10 mg/kg treatment. Scale bar: 100 μm. (I) The number of Ki‐67‐positive cells per 100 μm. (J) ZO‐1 intensity of DMSO‐treated BM. Scale bar: 100 μm. (K) ZO‐1 intensity of MMC‐treated BM (10 mg/kg). Scale bar: 100 μm. (L) Relative ZO‐1 to epithelial layers.

To exclude the effects of inflammation on ZO‐1 distribution, we intraperitoneally administered mitomycin C (MMC), which reduces cell proliferation by inhibiting DNA replication, in C57BL/6 mice (Figure 2A). The body weight reduction due to MMC treatment was small and not statistically significant (Figure 2B). MMC treatment reduced the thickness of oral mucosal epithelium in a dose‐dependent manner (Figure 2C–F), as well as the number of Ki‐67‐positive basal keratinocytes (Figure 2G–I), confirming epithelial hypoproliferation in the treated mice. In line with OLP samples, ZO‐1 showed a more limited dispersal pattern in MMC‐treated oral mucosal epithelium (10 mg/mL; Figure 2J–L). These data demonstrate that slower proliferation and turnover lead to less dispersed ZO‐1 distribution in the epithelium.

### 3.3 Dispersed ZO‐1 in human psoriatic skin

**FIGURE 3:**
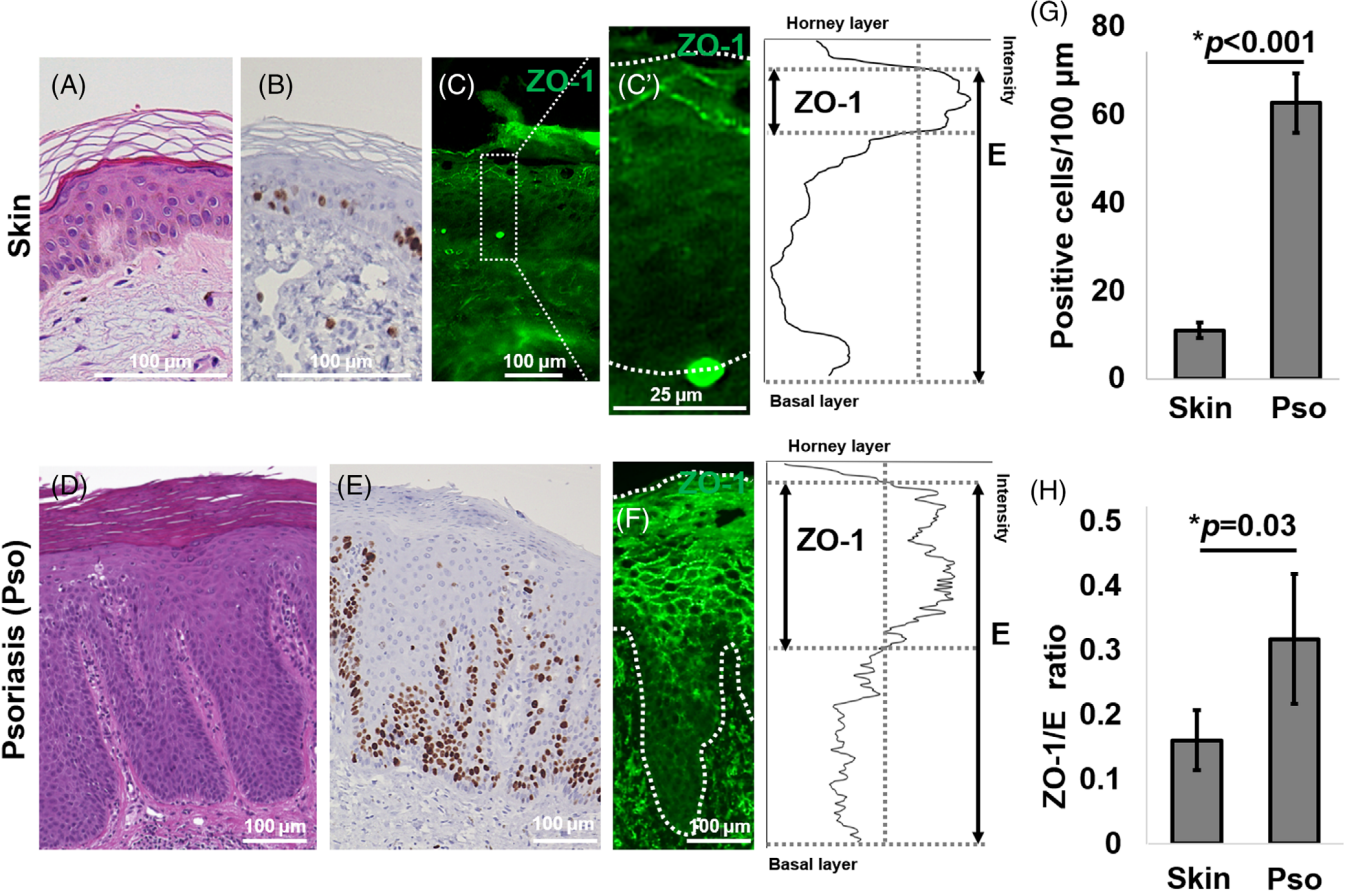
Zonula occludens‐1 (ZO‐1) distribution in human psoriatic skin. (A) HE staining of normal human skin. Scale bar: 100 μm. (B) Ki‐67 staining of normal human skin. Scale bar: 100 μm. (C) ZO‐1 immunofluorescent staining of normal human skin. Scale bar: 100 μm. (C’) Left: the enlarged image of the white square of (C). Scale bar: 25 μm. Right: ZO‐1 intensity of the left panel was plotted using the image J. (D) HE staining of psoriatic skin. Scale bar: 100 μm. (E) Ki‐67 staining of psoriatic skin. Scale bar: 100 μm. (F) Left: ZO‐1 immunofluorescent staining. Scale bar: 100 μm. Right: ZO‐1 intensity. (G) The number of Ki‐67‐positive cells per 100 μm. (H) Relative ZO‐1 area to epithelial layers.

Based on the results in mouse skin, we speculated whether faster proliferation and turnover altered ZO‐1 distributions in human psoriatic skin. Psoriasis is an inflammatory skin disease that is hyperproliferative in the epidermis and shortens the epidermal turnover period to 4–5 days,^34^ compared to 40–56 days^32^ in normal skin. The psoriatic epidermis is thicker than that of normal human epidermis (410 vs. 52 μm, on average; Figure 3A,D), and we found that Ki‐67‐positive cells were more abundant in psoriatic epidermis compared to normal skin (Figure 3B,E,G). Moreover, ZO‐1 was confined to 1–2 layers of the upper epidermis in normal human skin (Figure 3C’) as described previously.^18^ In contrast, ZO‐1 was distributed broadly in the psoriatic epidermis (Figure 3F,H), simulating the distribution pattern found in normal human buccal mucosa (Figure 1C,H). Similar to ZO‐1, occludin was present in multiple epithelial layers in the psoriatic epidermis. In contrast, claudin‐1 was distributed through the epidermis in both the normal human skin and the psoriatic skin (Figure S3).

### 3.4 Dispersal of ZO‐1 in murine psoriasis‐like skin

**FIGURE 4:**
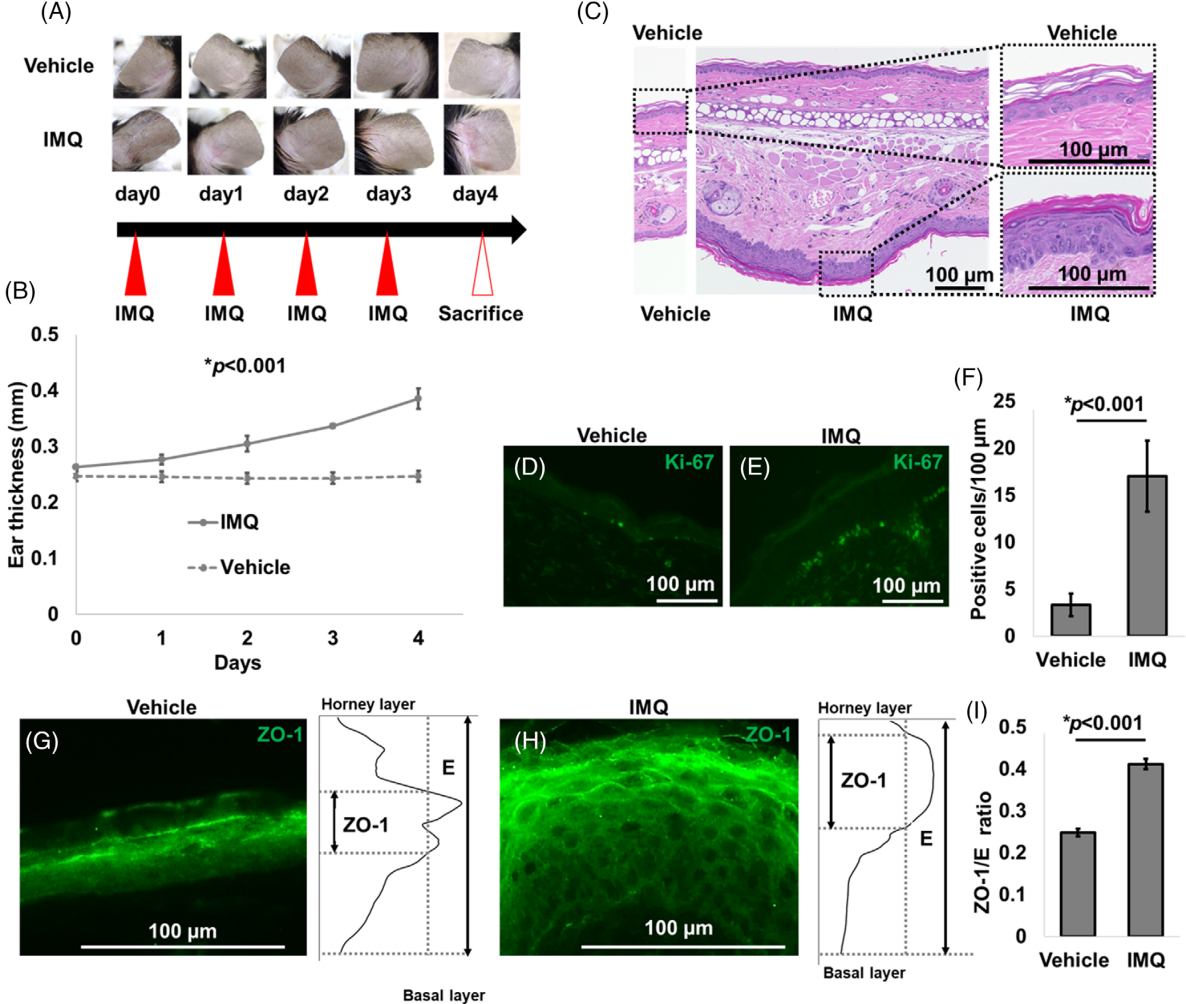
Zonula occludens‐1 (ZO‐1) distribution in murine psoriatic skin. (A) Schematic diagram of IMQ treatment and clinical manifestations of vehicle‐ or IMQ‐treated ear skin. (B) The ear thickness of vehicle‐ or IMQ‐treated skin. (C) HE staining of vehicle‐ or IMQ‐treated ear skin. Scale bar: 100 μm. (D) Ki‐67 immunofluorescent staining of vehicle‐treated skin. Scale bar: 100 μm. (E) Ki‐67 immunofluorescent staining of IMQ‐treated skin. Scale bar: 100 μm. (F) The number of Ki‐67‐positive cells per 100 μm. (G) ZO‐1‐labelled skin with vehicle treatment and ZO‐1 intensity. (H) ZO‐1‐labelled skin with IMQ treatment and ZO‐1 intensity. (I) Relative ZO‐1 area to epithelial layers.

To further confirm the spatial alteration of ZO‐1 in skin under conditions of faster proliferation and turnover, we investigated murine psoriasis‐like ear skin induced by topical IMQ application^20^ (Figure 4A–I). IMQ‐treated ears gradually developed scaly erythema (Figure 4A), and IMQ‐treated epidermis was significantly thicker and more hyperproliferative than that in vehicle‐treated controls (Figure 4B–F). In line with human psoriatic skin samples (Figure 3F,H), ZO‐1 was more broadly distributed in the IMQ‐treated epidermis (Figure 4G–I). These data on human and murine psoriasis indicate that faster proliferation and turnover result in a broader distribution of ZO‐1 in the skin.

### 3.5 Mathematical modelling

**FIGURE 5:**
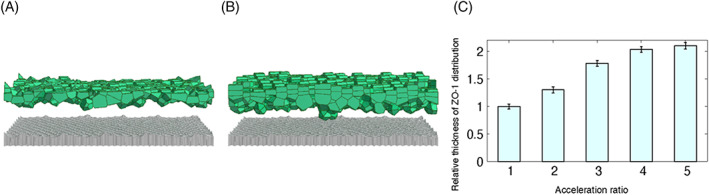
Simulation of ZO‐1 distribution. (A,B) Vertical section of the simulated epidermis. Epidermal cells are placed on the basement membrane (grey). Only SG2 cells (green), which express ZO‐1, are shown among the stratified epidermal cells. The Voronoi partition is used for the visualization of the cells. (A) Control and (B) proliferation and turnover four times faster than the control. (C) Relative thickness of ZO‐1 distribution as a function of the acceleration ratio *λ*, where proliferation and turnover are made *λ* times faster than the control, normalized by the thickness of the control (*λ* = 1). For each *λ*, the thickness was averaged over time.

The results from the human, mouse and cultured cell experiments led us to speculate whether epithelial cell proliferation and turnover are sufficient to determine ZO‐1 distribution. To answer this question, we employed mathematical modelling and a simulator of the epidermis^30^ to compute ZO‐1 distribution by varying the periods of proliferation and turnover. Compared to the control (Figure 5A), ZO‐1 distribution was broader when proliferation and turnover were accelerated (Figure 5B). Moreover, the thickness of the ZO‐1 distribution increased as the acceleration rate increased (Figure 5C). These results suggest that the acceleration of cell proliferation and turnover can lead to a broadening of ZO‐1 distribution.

### 3.6 Relative decrease in ZO‐1 and attenuation of barrier function upon acceleration of keratinocyte proliferation

**FIGURE 6:**
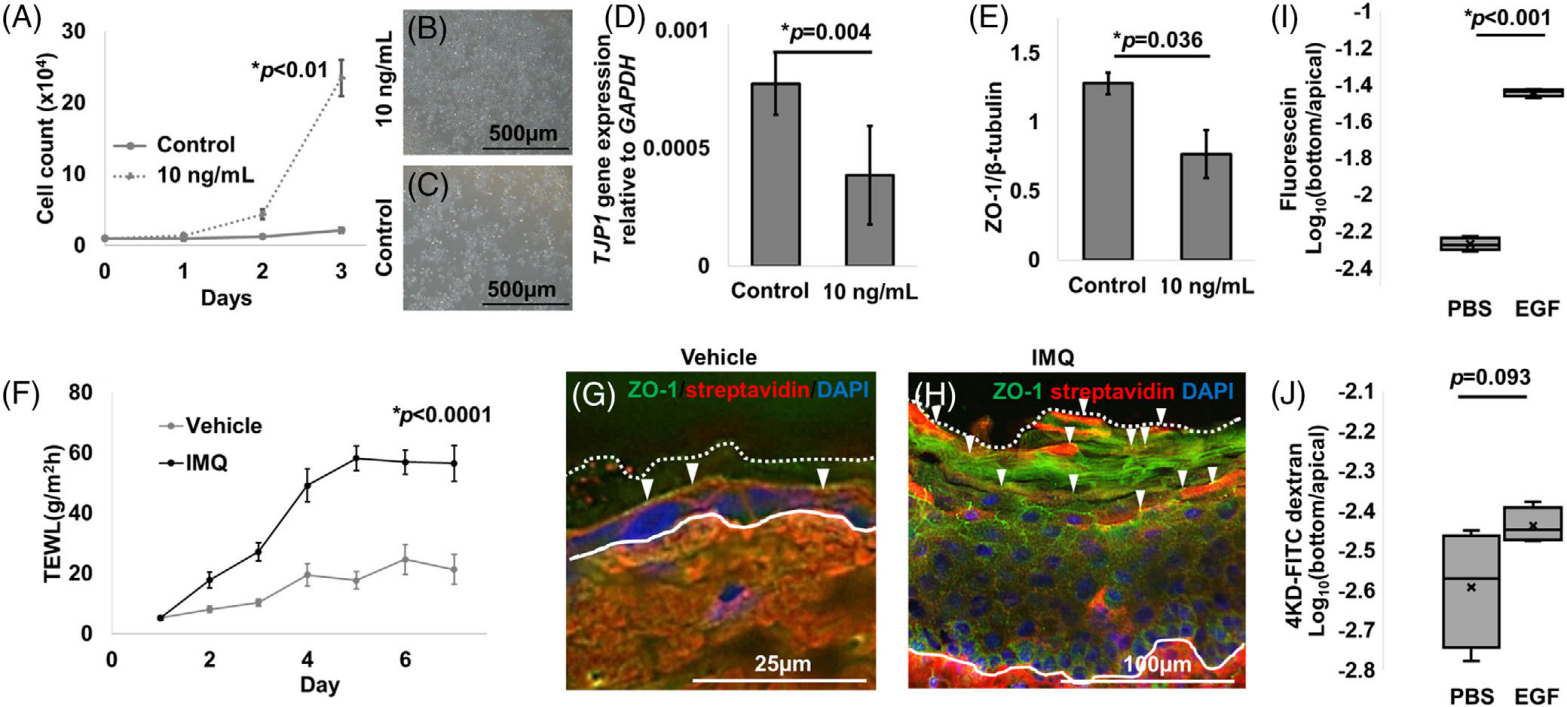
Barrier function of EGF‐treated cultured keratinocytes and murine psoriatic skin. (A) Cell proliferation curve of KerCT cells upon EGF treatment. (B) KerCT cells treated without EGF in the bright field. Scale Bar: 500 μm. (C) KerCT cells treated with EGF 10 ng/mL in the bright field. Scale Bar: 500 μm. (D) *TJP1* qPCR upon EGF treatment. (E) ZO‐1 protein level upon EGF treatment. (F) TEWL analysis of murine psoriatic skin. (G,H) A biotin tracer assay using murine ear skin. Control (G) and murine psoriatic skin (H). White arrowheads: tracer diffusion blockade. White line: basement membrane, white dotted line: outermost layer of the stratum corneum. Scale bar: 25 μm. (I,J) Paracellular flux assay. Leakage of fluorescein (I) and 4 kDa FITC‐dextran (J).

We subsequently explored whether ZO‐1 was quantitatively affected by cellular dynamics. EGF is a strong inducer of cultured keratinocyte proliferation^35^ (Figure 6A–C). In contrast, gene expression of *TJP1*, encoding ZO‐1, was reduced after EGF treatment (Figure 6D). ZO‐1 protein was also decreased in the treated epidermal cell sheet (Figure 6E). Transepidermal water loss (TEWL) measurement and biotin tracer assay revealed a barrier dysfunction in the IMQ mice(Figure 6F–H). Similarly, the SEM treated with EGF showed the apical leakage of biotin tracer as well as the reduction in ZO‐1 expression (Figure S4a–f). We further evaluated the outside‐in barrier by applying fluorescein and 4‐kDa FITC dextran to the monolayer cell sheets (*N* = 6). Dye leakage from the apical side to the basal side was estimated. Fluorescein leaked more dye when EGF was added, whereas 4‐kDa FITC‐dextran showed no significant dye leakage in the EGF‐treated cells (Figure 6I,J). Although no significant differences were seen between the EGF‐treated groups and the controls at 24 h after incubation, TER was significantly higher with EGF administration after 2 weeks when multi‐layered epidermis was present (data not shown). These data indicate that ZO‐1 expression correlates negatively with cell proliferation and that the barrier function is attenuated in hyperproliferative epithelia, as observed in normal buccal mucosa and psoriatic skin (Figures 1A–C, 3D–F, and 4H–I).

### 3.7 Expression of other epithelial and apoptosis markers in the epithelia with accelerated and slower cell proliferation/turnover

**FIGURE 7:**
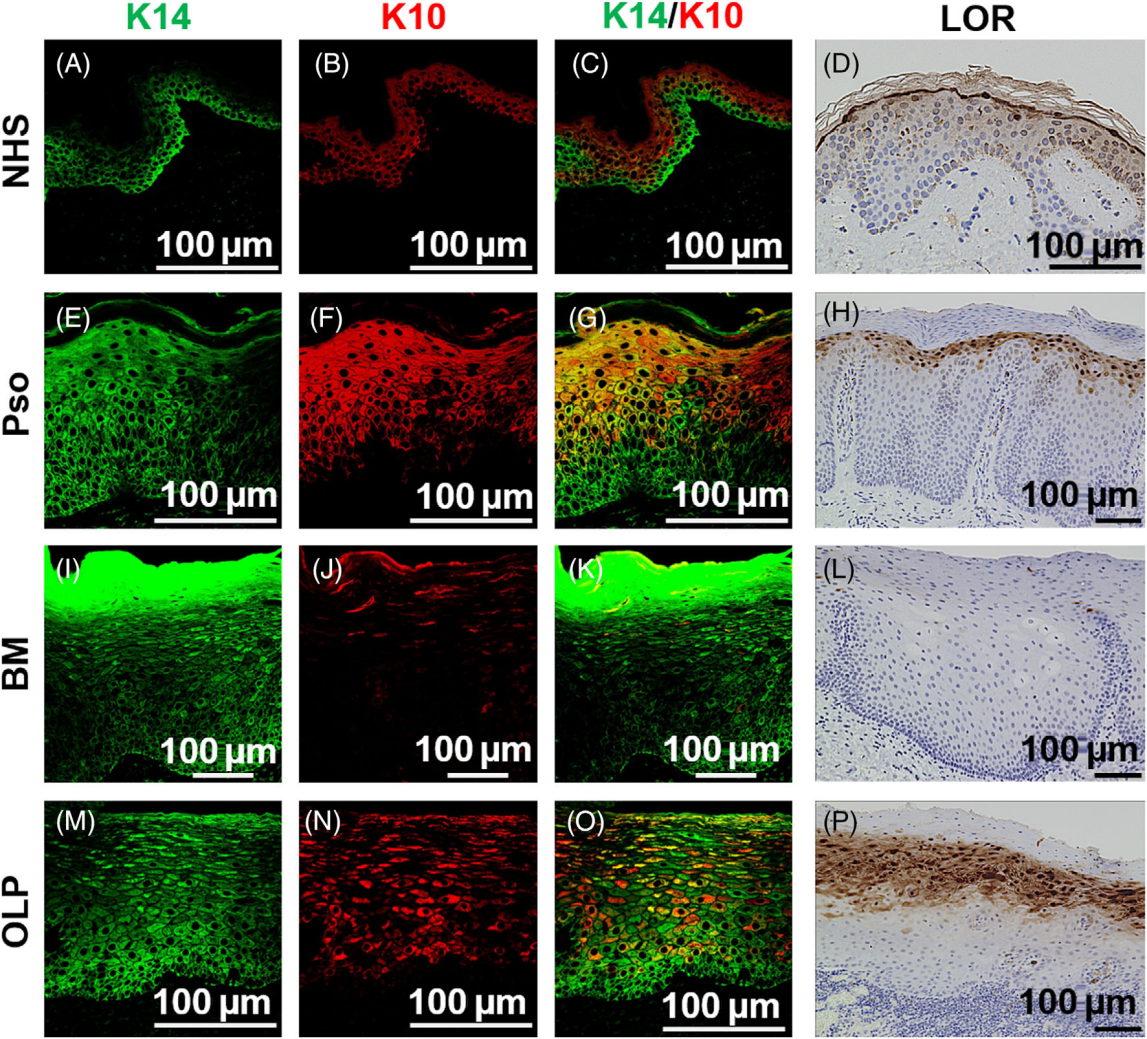
K14, K10 and Loricrin (LOR) staining of normal human skin (NHS), psoriatic skin (Pso), normal buccal mucosa (BM) and oral lichen planus (OLP). (A–D) K14 (A), K10 (B) and LOR (D) staining and a merged image (C) of NHS. Scale bar: 100 μm. (E–H) K14 (E), K10 (F) and LOR (H) staining and a merged image (G) of NHS. Scale bar: 100 μm. (I–L) K14 (I), K10 (J) and LOR (L) staining and a merged image (K) of NHS. Scale bar: 100 μm. (M–P) K14 (M), K10 (N) and LOR (P) staining and a merged image (O) of NHS. Scale bar: 100 μm.

Our findings demonstrate that ZO‐1 distribution correlates with cell proliferation/turnover, and yet, other epithelial markers may exhibit similar expression patterns. To test this hypothesis, we utilized keratin 14 (K14), keratin 10 (K10) and loricrin as makers of basal, spinous and upper spinous layer cells, respectively (Figure 7A–P). K14 labelling was pronounced in basal keratinocytes of NHS, whereas this tendency was not obvious in the psoriatic, BM or OLP samples (Figure 7A,E,I,M). K10 labelling was present in the suprabasal layers in the NHS, psoriatic and OLP samples, but was greatly reduced in the BM (Figure 7B,F,J,N). Loricrin expression was restricted to the uppermost spinous layer of NHS and was dispersed across several layers in psoriasis and BM samples (Figure 7D,H,l). In contrast, loricrin was absent in OLP (Figure 7P). These data suggest that the expression of major epithelial markers may partially reflect epithelial proliferation/turnover rate, and yet, ZO‐1 may serve as a superior marker in this regard. We further excluded the involvement of apoptosis in ZO‐1 distribution through the assessment of cleaved caspase‐3 (CCP3) labelling, which showed that CCP3‐positive cells were present in the psoriatic samples,^36^ while NHS, BM and OLP had limited or no presence of such cells (Figure S5).

## 4 DISCUSSION

This study examined the role of cell proliferation and turnover in determining the distribution patterns of ZO‐1. Slower epithelial proliferation and turnover (e.g., normal skin, OLP and MMC‐treated mucosa) resulted in a restricted distribution ZO‐1, whereas faster proliferation and turnover (e.g., normal buccal mucosa and psoriasis) increased the distribution of ZO‐1 in the epithelium. It is noteworthy that this altered distribution indirectly relates to cell proliferation rates, as proliferating cells do not express ZO‐1.

Cell proliferation and turnover are closely linked processes and are often distorted into a faster state, such as in psoriasis^37^, ^38^ or a slower state, as in OLP.^33^ One caveat of our study is that we could not solely manipulate proliferation or turnover without affecting the other. Further studies are needed to elucidate the contribution of each of these factors to the dynamics of ZO‐1.

In murine ear skin, TJs are located in the SG2 layer where they form a flat barrier, and their distribution is strictly controlled by dynamic remodelling. Based on our results, epithelia with faster proliferation and turnover form continuous ZO‐1 over several layers (Figure S2). In epithelia with these characteristics, the cells may start expressing ZO‐1 earlier in the differentiation step to functionally compensate for immature junctional complexes. In line with this scenario, when we investigated a single cell, ZO‐1 did not cover the whole cell periphery in the epithelia with faster proliferation and turnover (Figure S2). However, it is still possible that the production and degradation of ZO‐1 protein (protein turnover) are altered with the cell proliferation status. Long‐term live imaging may solve this issue in future studies.

Previous studies have shown that TEWL is higher in psoriatic skin than in normal skin,^39^, ^40^ which is consistent with our data (Figure 6F). This barrier dysfunction has been attributed to the disruption of epidermal tight, gap and adhesion‐binding proteins.^41^ In normal epidermis, TJ proteins are expressed only in the granular and upper spinous layers. However, in psoriatic lesions, the TJ proteins, including ZO‐1, occludin and claudin‐4, are highly expressed outside the granular layer^42^, ^43^ (Figure S3d–f) and are downregulated, suggesting an overall impairment of the TJ function.^44^, ^45^ This impairment is likely to contribute to the increased TEWL and decreased hydration seen in psoriatic lesions.^46^ IL‐17 and IL‐1β, which are implicated in psoriasis pathogenesis, are also known to downregulate keratinocyte adhesion proteins, at least partially explaining the dysregulation of the TJ function in psoriasis.^47^, ^48^ Our study confirms the barrier dysfunction in the psoriatic epidermis and suggests that proliferation and turnover may be responsible for this dysfunction.

Our study took advantage of OLP for hypoproliferative conditions in the mucosal epithelium. Previous studies have shown the expression of TJ proteins in cutaneous lichen planus (CLP) and OLP^49,50^; although to our knowledge, ZO‐1 labelling in OLP has not been investigated. ZO‐1 labelling in CLP was described in one paper as being as extensive as in psoriatic epidermis.^49^ However, upon reexamination of the original figure, it appears that ZO‐1 immunoreactivity in CLP was weak and its distribution was restricted.^49^

In conclusion, the confinement of ZO‐1 within a single layer of squamous epithelia is indicative of slow epithelial proliferation and turnover rates. Our findings do not fully elucidate the functional significance of ZO‐1 distribution, but our study does suggest that ZO‐1 reflects the proliferation and turnover of epithelia, which could have potential use as a marker of disease severity in psoriasis and OLP. Among the TJ proteins, claudin‐1 was unable to discriminate between epidermis from normal human subjects and psoriasis patients (Figure S3). Occludin exhibited a similar expression pattern to ZO‐1 (Figure S3), but occludin signals appeared as discrete dots in the images. Therefore, ZO‐1 labelling may be a more suitable proxy for this purpose. Further studies utilizing clinical samples and live imaging are needed to confirm our hypothesis.

## AUTHOR CONTRIBUTIONS

Keisuke Imafuku, Hiroaki Iwata and Ken Natsuga have made substantioal contributions to conception and design or acquisition, analysis and interpretation of data. Makoto Okumura, Yasuaki Kobayashi and Masaharu Nagayama contributed to create matnmatical model. Hiroyuki Kitahata provided us image J plugins and contributed to analysis of data. Akiharu Kubo involved in revising the manuscript critically for important intellectual content. Hideyuki Ujiie Given final approval of the version to be published. All authors have participated sufficiently in the work to take public responsibility for appropriate portions of the content and agreed to be accountable for all aspects of the work in ensuring that questions related to the accuracy or integrity of any part of the work are appropriately investigated and resolved.

## FUNDING INFORMATION

JSPS Grant‐in‐Aid for Scientific Research (C) #19K08786 to Hiroaki Iwata. The Nakatomi Foundation to Hiroaki Iwata JSID's Fellowship Shiseido Research Grant 2020 and KOSÉ Cosmetology Research Foundation to Hiroaki Iwata. The Mochida Memorial Foundation for Medical and Pharmaceutical Research to Ken Natsuga. JST, CREST Grant Number JPMJCR1926 to Masaharu Nagayama and Makoto Okumura. JSPS KAKENHI Grant Number JP19K03629 to Yasuaki Kobayashi.

## CONFLICT OF INTEREST STATEMENT

We declare that we have no conflicts of interest.

## INFORMED CONSENT

Participants or their legal guardians provided written informed consent.

## Supporting information


**Data S1:** Supporting Information

## Data Availability

Data sharing is not applicable to this article as no new data were created or analyzed in this study.

## References

[1] Natsuga K . Epidermal barriers. Cold Spring Harb Perspect Med. 2014;4:a018218. 24692192 10.1101/cshperspect.a018218 PMC3968788

[2] Farquhar MG , Palade GE . Junctional complexes in various epithelia. J Cell Biol. 1963;17:375‐412. 13944428 10.1083/jcb.17.2.375 PMC2106201

[3] Zihni C , Mills C , Matter K , Balda MS . Tight junctions: from simple barriers to multifunctional molecular gates. Nat Rev Mol Cell Biol. 2016;17:564‐580. 27353478 10.1038/nrm.2016.80

[4] Schneeberger EE , Lynch RD . The tight junction: a multifunctional complex. Am J Physiol Cell Physiol. 2004;286:C1213‐C1228. 15151915 10.1152/ajpcell.00558.2003

[5] Yokouchi M , Atsugi T , van Logtestijn M , et al. Epidermal cell turnover across tight junctions based on Kelvin's tetrakaidecahedron cell shape. Elife. 2016;5:e19593. 27894419 10.7554/eLife.19593 PMC5127639

[6] Yoshida K , Yokouchi M , Nagao K , Ishii K , Amagai M , Kubo A . Functional tight junction barrier localizes in the second layer of the stratum granulosum of human epidermis. J Dermatol Sci. 2013;71:89‐99. 23712060 10.1016/j.jdermsci.2013.04.021

[7] Anderson JM , Van Itallie CM . Physiology and function of the tight junction. Cold Spring Harb Perspect Biol. 2009;1:a002584. 20066090 10.1101/cshperspect.a002584 PMC2742087

[8] Furuse M , Hirase T , Itoh M , et al. OCCLUDIN—a novel integral membrane‐protein localizing AT tight junctions. J Cell Biol. 1993;123:1777‐1788. 8276896 10.1083/jcb.123.6.1777 PMC2290891

[9] Ikenouchi J , Furuse M , Furuse K , Sasaki H , Tsukita S , Tsukita S . Tricellulin constitutes a novel barrier at tricellular contacts of epithelial cells. J Cell Biol. 2005;171:939‐945. 16365161 10.1083/jcb.200510043 PMC2171318

[10] Higashi T , Tokuda S , Kitajiri S , et al. Analysis of the ‘angulin’ proteins LSR, ILDR1 and ILDR2—tricellulin recruitment, epithelial barrier function and implication in deafness pathogenesis. J Cell Sci. 2013;126:966‐977. 23239027 10.1242/jcs.116442

[11] Bazzoni G . The JAM family of junctional adhesion molecules. Curr Opin Cell Biol. 2003;15:525‐530. 14519386 10.1016/s0955-0674(03)00104-2

[12] Furuse M , Hata M , Furuse K , et al. Claudin‐based tight junctions are crucial for the mammalian epidermal barrier: a lesson from claudin‐1‐deficient mice. J Cell Biol. 2002;156:1099‐1111. 11889141 10.1083/jcb.200110122 PMC2173463

[13] Kubota K , Furuse M , Sasaki H , et al. Ca(2+)‐independent cell‐adhesion activity of claudins, a family of integral membrane proteins localized at tight junctions. Curr Biol. 1999;9:1035‐1038. 10508613 10.1016/s0960-9822(99)80452-7

[14] Furuse M , Sasaki H , Fujimoto K , Tsukita S . A single gene product, claudin‐1 or ‐2, reconstitutes tight junction strands and recruits occludin in fibroblasts. J Cell Biol. 1998;143:391‐401. 9786950 10.1083/jcb.143.2.391 PMC2132845

[15] Stevenson BR , Siliciano JD , Mooseker MS , Goodenough DA . Identification of ZO‐1: a high molecular weight polypeptide associated with the tight junction (zonula occludens) in a variety of epithelia. J Cell Biol. 1986;103:755‐766. 3528172 10.1083/jcb.103.3.755 PMC2114282

[16] González‐Mariscal L , Betanzos A , Avila‐Flores A . MAGUK proteins: structure and role in the tight junction. Semin Cell Dev Biol. 2000;11:315‐324. 10966866 10.1006/scdb.2000.0178

[17] Ikenouchi J , Umeda K , Tsukita S , Furuse M , Tsukita S . Requirement of ZO‐1 for the formation of belt‐like adherens junctions during epithelial cell polarization. J Cell Biol. 2007;176:779‐786. 17353356 10.1083/jcb.200612080 PMC2064052

[18] Matsumoto K , Furuya T , Tobe M . Effect of mitomycin C on bone marrow cells in mice. J Toxicol Sci. 1984;9:51‐56. 6433039 10.2131/jts.9.51

[19] Gilliet M , Conrad C , Geiges M , et al. Psoriasis triggered by toll‐like receptor 7 agonist imiquimod in the presence of dermal plasmacytoid dendritic cell precursors. Arch Dermatol. 2004;140:1490‐1495. 15611427 10.1001/archderm.140.12.1490

[20] Kamaguchi M , Iwata H , Ujiie H , et al. High expression of collagen XVII compensates for its depletion induced by pemphigoid IgG in the oral mucosa. J Invest Dermatol. 2018;138:1707‐1715. 29530535 10.1016/j.jid.2018.03.002

[21] Imafuku K , Kamaguchi M , Natsuga K , Nakamura H , Shimizu H , Iwata H . Zonula occludens‐1 demonstrates a unique appearance in buccal mucosa over several layers. Cell Tissue Res. 2021;384:691‐702. 33635425 10.1007/s00441-021-03425-8

[22] Kubo A , Nagao K , Amagai M . 3D visualization of epidermal Langerhans cells. Methods Mol Biol. 2013;961:119‐127. 23325638 10.1007/978-1-62703-227-8_5

[23] Fujimura Y , Watanabe M , Ohno K , et al. Hair follicle stem cell progeny heal blisters while pausing skin development. EMBO Rep. 2021;22:e50882. 34085753 10.15252/embr.202050882 PMC8256293

[24] Smits JPH , Niehues H , Rikken G , et al. Immortalized N/TERT keratinocytes as an alternative cell source in 3D human epidermal models. Sci Rep. 2017;7:11838. 28928444 10.1038/s41598-017-12041-y PMC5605545

[25] Chen S , Einspanier R , Schoen J . Transepithelial electrical resistance (TEER): a functional parameter to monitor the quality of oviduct epithelial cells cultured on filter supports. Histochem Cell Biol. 2015;144:509‐515. 26223877 10.1007/s00418-015-1351-1 PMC4628619

[26] Yokouchi M , Kubo A , Kawasaki H , et al. Epidermal tight junction barrier function is altered by skin inflammation, but not by filaggrin‐deficient stratum corneum. J Dermatol Sci. 2015;77:28‐36. 25511077 10.1016/j.jdermsci.2014.11.007

[27] Chen Y , Merzdorf C , Paul DL , Goodenough DA . COOH terminus of occludin is required for tight junction barrier function in early Xenopus embryos. J Cell Biol. 1997;138:891‐899. 9265654 10.1083/jcb.138.4.891 PMC2138038

[28] Otani T , Nguyen TP , Tokuda S , et al. Claudins and JAM‐A coordinately regulate tight junction formation and epithelial polarity. J Cell Biol. 2019;218:3372‐3396. 31467165 10.1083/jcb.201812157 PMC6781433

[29] Ohno K , Kobayashi Y , Uesaka M , et al. A computational model of the epidermis with the deformable dermis and its application to skin diseases. Sci Rep. 2021;11:13234. 34168195 10.1038/s41598-021-92540-1 PMC8225835

[30] Cutright DE , Bauer H . Cell renewal in the oral mucosa and skin of the rat. I. Turnover time. Oral Surg Oral Med Oral Pathol. 1967;23:249‐259. 5225664 10.1016/0030-4220(67)90104-1

[31] Halprin KM . Epidermal “turnover time”—a re‐examination. Br J Dermatol. 1972;86:14‐19. 4551262 10.1111/j.1365-2133.1972.tb01886.x

[32] Lehman JS , Tollefson MM , Gibson LE . Lichen planus. Int J Dermatol. 2009;48:682‐694. 19570072 10.1111/j.1365-4632.2009.04062.x

[33] Lowes MA , Suárez‐Fariñas M , Krueger JG . Immunology of psoriasis. Annu Rev Immunol. 2014;32:227‐255. 24655295 10.1146/annurev-immunol-032713-120225 PMC4229247

[34] Cohen S . Isolation of a mouse submaxillary gland protein accelerating incisor eruption and eyelid opening in the new‐born animal. J Biol Chem. 1962;237:1555‐1562. 13880319

[35] Bebars SMM , Al‐Sharaky DR , Gaber MA , et al. Immunohistochemical expression of Caspase‐3 in psoriasis. J Clin Diagn Res. 2017;11:Ec01‐Ec05. 10.7860/JCDR/2017/25609.10145 PMC5583936 28892900

[36] Li J , Li X , Hou R , et al. Psoriatic T cells reduce epidermal turnover time and affect cell proliferation contributed from differential gene expression. J Dermatol. 2015;42:874‐880. 26046687 10.1111/1346-8138.12961

[37] Beutner KR , Tyring S . Human papillomavirus and human disease. Am J Med. 1997;102:9‐15. 10.1016/s0002-9343(97)00178-2 9217657

[38] Takahashi H , Tsuji H , Minami‐Hori M , Miyauchi Y , Iizuka H . Defective barrier function accompanied by structural changes of psoriatic stratum corneum. J Dermatol. 2014;41:144‐148. 24471458 10.1111/1346-8138.12393

[39] Nikam VN , Monteiro RC , Dandakeri S , Bhat RM . Transepidermal water loss in psoriasis: a case‐control study. Indian Dermatol Online J. 2019;10:267‐271. 31149569 10.4103/idoj.IDOJ_180_18 PMC6536057

[40] Orsmond A , Bereza‐Malcolm L , Lynch T , March L , Xue M . Skin barrier dysregulation in psoriasis. Int J Mol Sci. 2021;22:10841. 34639182 10.3390/ijms221910841 PMC8509518

[41] Peltonen S , Riehokainen J , Pummi K , Peltonen J . Tight junction components occludin, ZO‐1, and claudin‐1, ‐4 and ‐5 in active and healing psoriasis. Br J Dermatol. 2007;156:466‐472. 17300235 10.1111/j.1365-2133.2006.07642.x

[42] Kirschner N , Poetzl C , von den Driesch P , et al. Alteration of tight junction proteins is an early event in psoriasis: putative involvement of proinflammatory cytokines. Am J Pathol. 2009;175:1095‐1106. 19661441 10.2353/ajpath.2009.080973 PMC2731128

[43] Wang X , Liu X , Liu N , Chen H . Prediction of crucial epigenetically‐associated, differentially expressed genes by integrated bioinformatics analysis and the identification of S100A9 as a novel biomarker in psoriasis. Int J Mol Med. 2020;45:93‐102. 31746348 10.3892/ijmm.2019.4392 PMC6889933

[44] Visconti B , Paolino G , Carotti S , et al. Immunohistochemical expression of VDR is associated with reduced integrity of tight junction complex in psoriatic skin. J Eur Acad Dermatol Venereol. 2015;29:2038‐2042. 25220655 10.1111/jdv.12736

[45] Montero‐Vilchez T , Segura‐Fernández‐Nogueras MV , Pérez‐Rodríguez I , et al. Skin barrier function in psoriasis and atopic dermatitis: transepidermal water loss and temperature as useful tools to assess disease severity. J Clin Med. 2021;10:359. 33477944 10.3390/jcm10020359 PMC7833436

[46] Watson RE , Poddar R , Walker JM , et al. Altered claudin expression is a feature of chronic plaque psoriasis. J Pathol. 2007;212:450‐458. 17582238 10.1002/path.2200

[47] Gutowska‐Owsiak D , Schaupp AL , Salimi M , et al. IL‐17 downregulates filaggrin and affects keratinocyte expression of genes associated with cellular adhesion. Exp Dermatol. 2012;21:104‐110. 22229441 10.1111/j.1600-0625.2011.01412.x

[48] Pummi K , Malminen M , Aho H , Karvonen SL , Peltonen J , Peltonen S . Epidermal tight junctions: ZO‐1 and occludin are expressed in mature, developing, and affected skin and in vitro differentiating keratinocytes. J Invest Dermatol. 2001;117:1050‐1058. 11710912 10.1046/j.0022-202x.2001.01493.x

[49] Hämäläinen L , Soini Y , Pasonen‐Seppänen S , Siponen M . Alterations in the expression of EMT‐related proteins claudin‐1, claudin‐4 and claudin‐7, E‐cadherin, TWIST1 and ZEB1 in oral lichen planus. J Oral Pathol Med. 2019;48:735‐744. 31228209 10.1111/jop.12917

