# Supplementary material for: Zonula occludens‐1 distribution and barrier functions are affected by epithelial proliferation and turnover rates
Source: Cell Prolif. 2023 Mar 14;56(9):e13441. doi: 10.1111/cpr.13441 (PMC10472521; doi:10.1111/cpr.13441)
Supplement: Supplementary file 1 — Data S1: Supporting Information [file CPR-56-e13441-s001.docx]

**Supplementary Table 1: Primer list.**

| *h:* *Homo sapiens* | Forward 5´→3´ | Reverse 5´→3´ |
| --- | --- | --- |
| *hTJP1* | AGAGGAAGCTGTGGGTAACG | AGGGTTTTCCTTGGCTGACA |
| *hGAPDH* | ACCATCTTCCAGGAGCGAGA | GACTCCACGACGTACTCAGC |

**
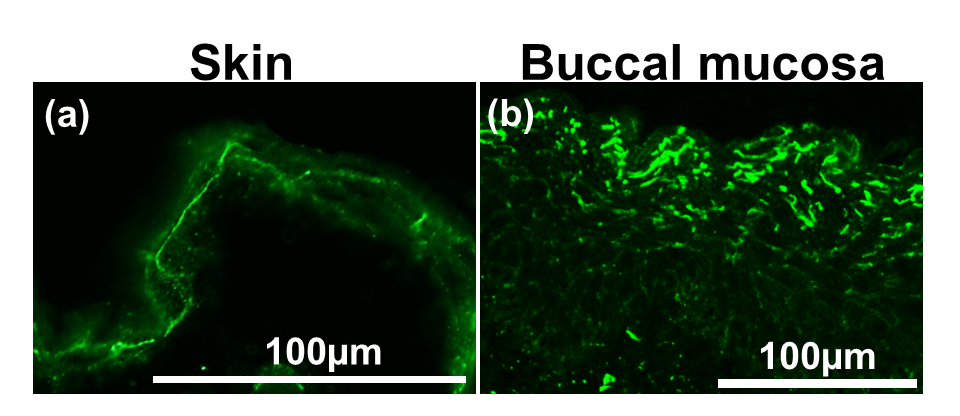
**

**Supplementary Figure 1. TJs in normal mouse skin and normal buccal mucosa^1^.**

(a) ZO-1 expression in the vertical section of a mouse skin sample. Scale bar: 100 µm.

(b) ZO-1 expression in the vertical section of a mouse buccal mucosa sample. Scale bar: 100 µm.

**
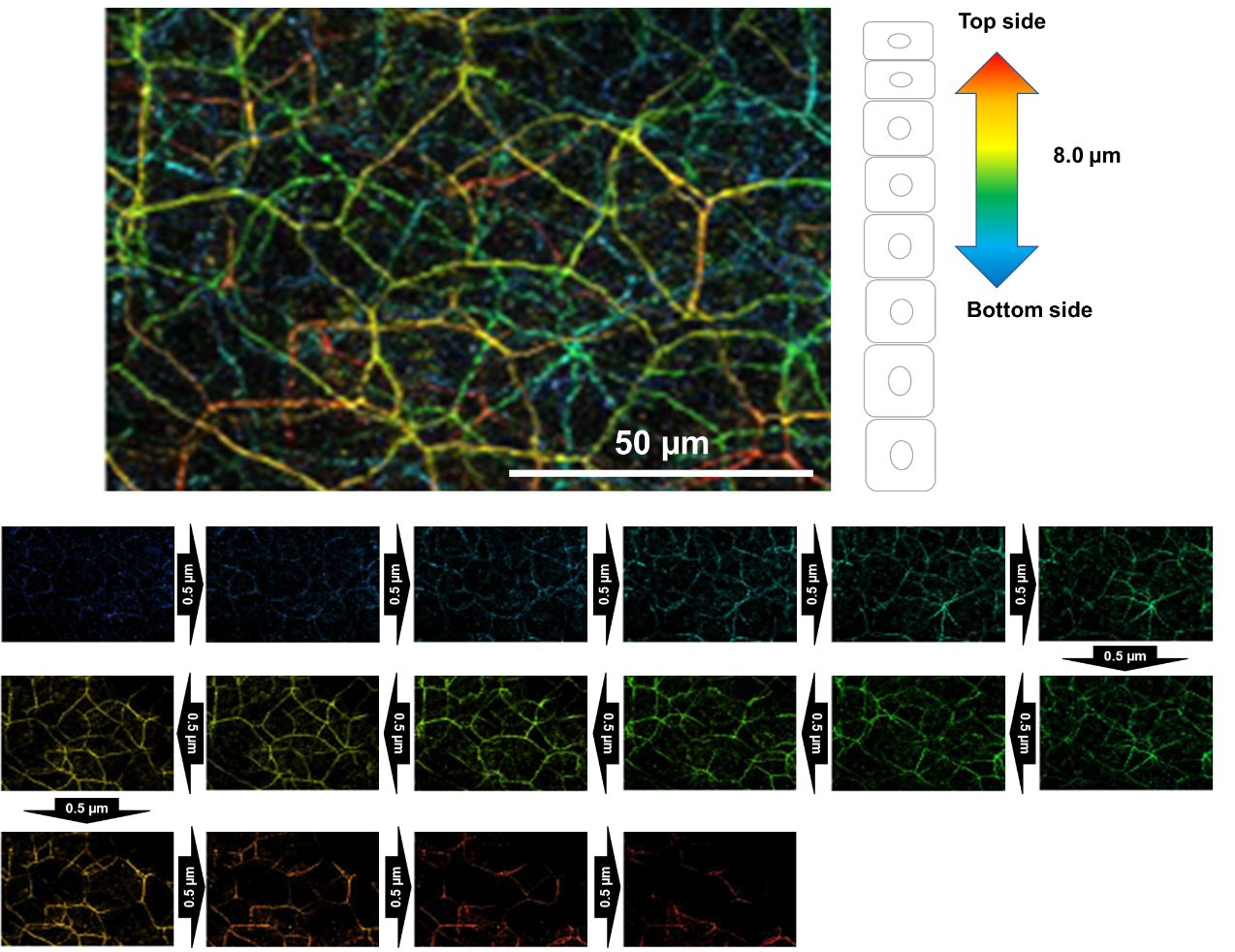
**

**Supplementary Figure 2: Whole-mount imaging of TJs in the buccal mucosa.**

Top: ZO-1 labeling of the whole-mount sample of mouse buccal mucosa. The pseudocolors indicate the distance between ZO-1 labeling and the mucosal surface. Scale bar: 50 µm, Bottom: ZO-1 labeling of the whole-mount sample. Color transformed and aligned every 0.5-µm slice from bottom to top.

**
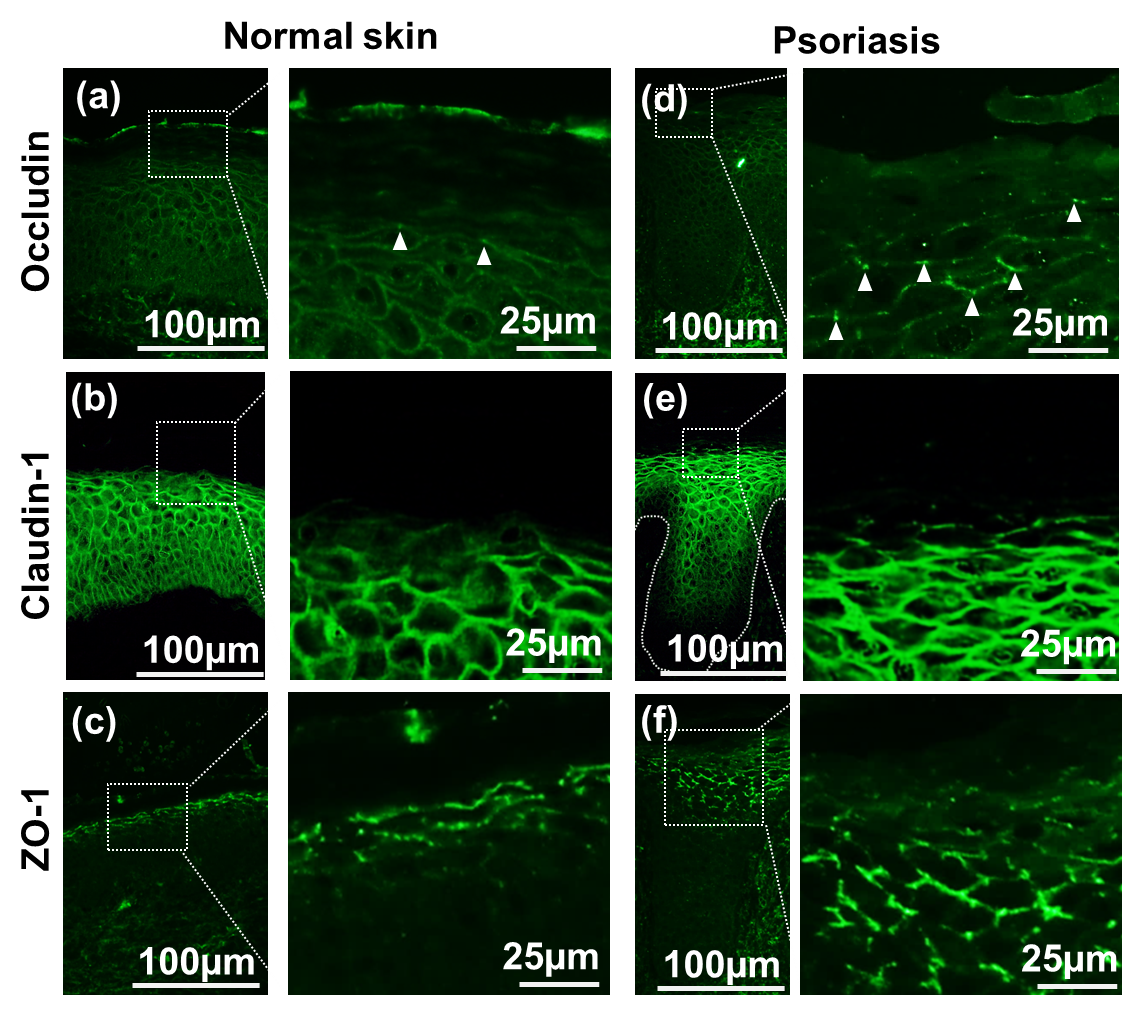
**

**Supplementary Figure 3: TJ-associated proteins in human psoriatic skin.**

(a-c) Occludin (a), Claudin-1 (b), and ZO-1 (c) staining of normal human skin. Left; low magnification. Scale bar: 100 µm. Right; high magnification of the white dotted rectangle. Scale bar: 25 µm.

(d-f) Occludin (d), Claudin-1 (e), and ZO-1 (f) staining of psoriatic skin. Left; low magnification. Scale bar: 100 µm. Right; high magnification of the white dotted rectangle. Scale bar: 25 µm.

**
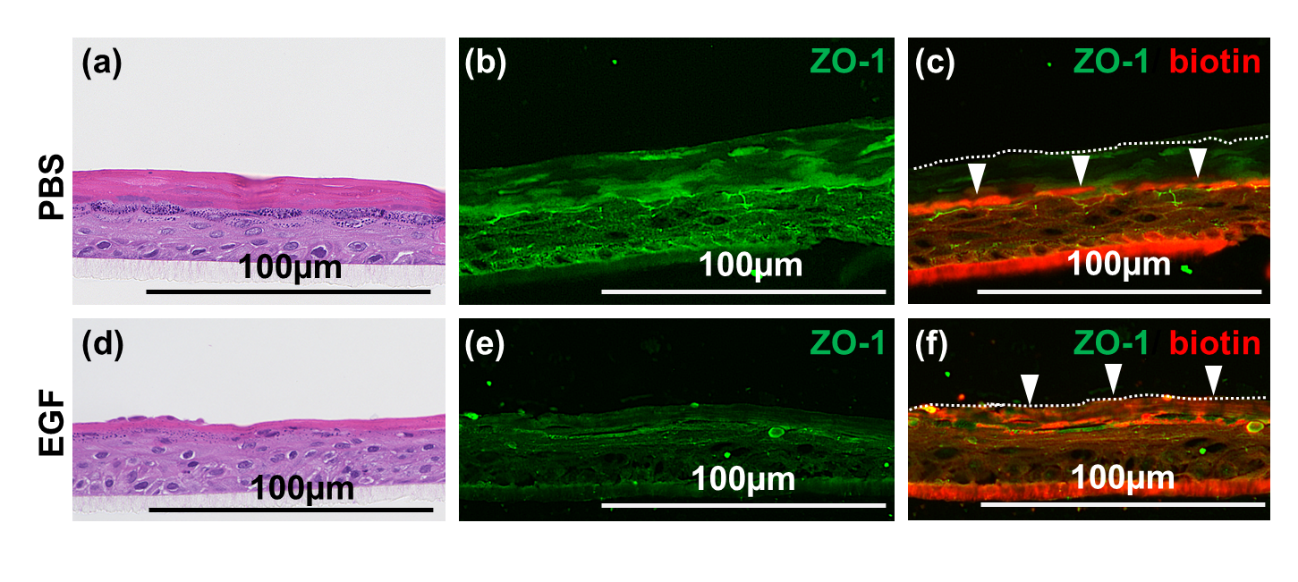
Supplementary Figure 4: Biotin tracer assay using skin equivalent model (SEM).**

1. HE staining of control SEM. Scale bar: 100 µm.
2. ZO-1 staining of control SEM. Scale bar: 100 µm.
3. ZO-1 and biotin tracer merged image of control SEM. White dotted line: apical side. Scale bar: 100 µm
4. HE staining of EGF-treated SEM. Scale bar: 100 µm.
5. ZO-1 staining of EGF-treated SEM. Scale bar: 100 µm.
6. ZO-1 and biotin tracer merged image of EGF-treated SEM. White dotted line: apical side. Scale bar: 100 µm

**
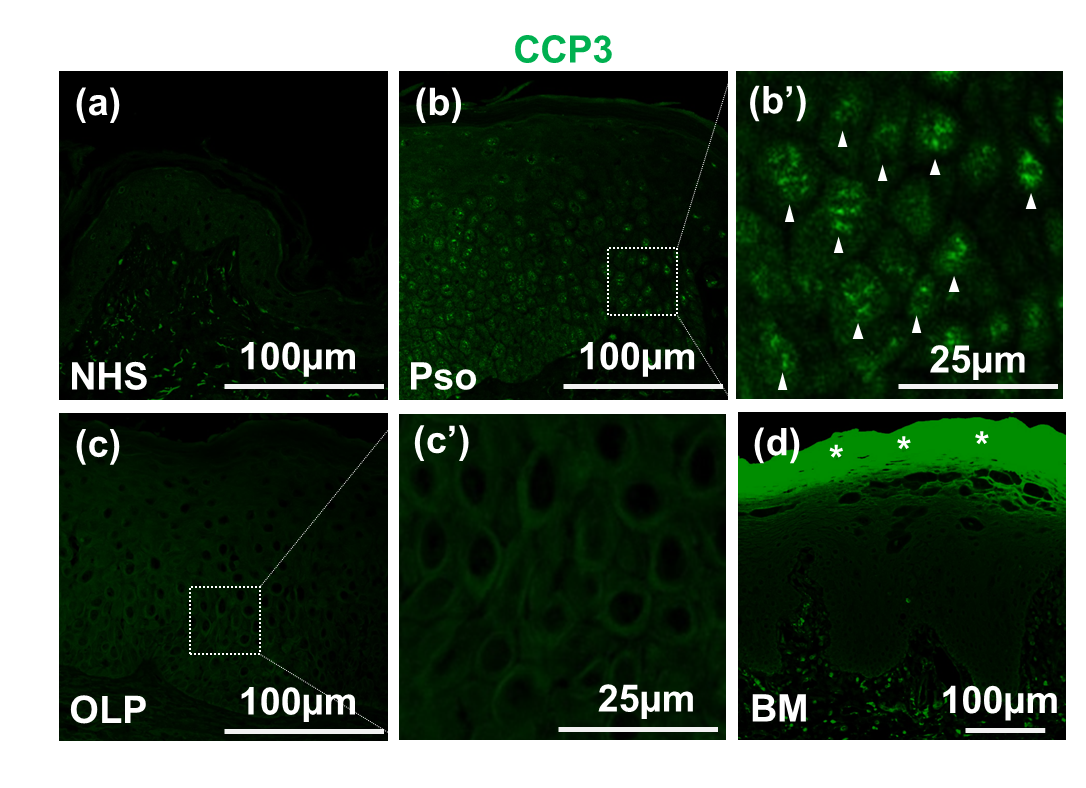
**

**Supplementary Figure 5. Cleaved-caspase 3 (CCP3) staining of normal human skin (NHS), psoriatic skin (Pso), normal buccal mucosa (BM), and oral lichen planus (OLP).**

(a) NHS, (b) Pso, (b’) high magnification, arrowheads: positive cells, (c) OLP, (c’) high magnification, and (d) BM, *nonspecific deposition, scale bar: 100 µm.

**References**

1 Imafuku K, Kamaguchi M, Natsuga K *et al.* Zonula occludens-1 demonstrates a unique appearance in buccal mucosa over several layers. *Cell Tissue Res* 2021; **384**: 691-702.
